# The expression of P16 and S100 associated with elastin degradation and fibrosis of the Ligamentum Flavum hypertrophy

**DOI:** 10.1186/s12891-019-2825-4

**Published:** 2019-10-22

**Authors:** Wei Hu, Shunli Kan, Guang Liu, Zegang Cao, Rusen Zhu

**Affiliations:** 1 Department of Spine Surgery, Tianjin Union Medical Center, Tianjin, 300121 China; 2 Department of Pathology, Tianjin Union Medical Center, Tianjin, 300121 China

**Keywords:** Ligamentum Flavum, Hypertrophy, Histology, Spinal stenosis, Elastin, Fibrosis, Magnetic resonance imaging

## Abstract

**Background:**

One of the characteristics of lumbar spinal stenosis (LSS) is elastin degradation and fibrosis in the ligamentum flavum (LF). However, the biochemical factors that cause these histologic changes is unclear. P16 and S100 participate in scar formation and collagen development in wound healing and fibrosis diseases. In this study, we investigate the association between P16 and S100 expression and the fibrosis of the hypertrophic LF in LSS.

**Methods:**

The LF specimens were surgically obtained from 30 patients with single-segment LSS (SLSS), 30 patients with double-segment LSS (DLSS) and 30 patients with L4/5 lumbar disc herniation (LDH). The LF thickness was measured by axial T1-weighted MRI. The extent of LF elastin degradation and fibrosis were graded based on hematoxylin-eosin (HE) and Verhoff’s Van Gieson’s (VVG) stain, respectively. The localization of P16 and S100 was determined by immunohistochemistry.

**Results:**

The Absolute and relative LF thickness were greater in the DLSS group compared with the SLSS and LDH groups (*p* <  0.05). The elastic tissue from the dorsal aspect to the dural aspect in SLSS and DLSS groups was significantly increased. The amount of collagen deposition and elastic tissue is significantly higher in the DLSS group compared with the SLSS and LDH groups (*p* <  0.05). The specimens in the DLSS group showed positive staining of P16, especially in the dorsal layer. Almost all samples in the SLSS group were partially positive for P16. The LDH group showed negative staining of P16 in both the dural and dorsal layers. All the three groups were stained with S100 in the dorsal layer of the LF. On the contrary, S100 staining was absent in the dural layer of the LF in the three groups.

**Conclusions:**

Elastin degradation and fibrosis of the LF in the DLSS patients is more severe compared with the SLSS and LDH patients. Increased expression of P16 associated with LF fibrosis and thickness, suggested that the expression of P16 may related to LF hypertrophy in the patients who suffer with LSS. LF hypertrophy process may not be associated with high expression of S100.

## Background

Lumbar spinal stenosis (LSS) is a common disease, usually causing lower back pain and limited walking. Patients with severe symptoms usually require operation. The causes of LSS include disc herniation, articular process hyperplasia, hypertrophy of the ligamentum flavum (LF) and lumbar instability [1, 2]. Intraoperative removal of the hypertrophic LF can usually achieve satisfactory clinical results [3, 4]. Experts have done a lot of biomechanic, cytologic and genetics studies on the LF. However, the mechanism of hypertrophy of the LF remains unclear [5–7]. It was shown in recent study that applying consecutive mechanical flexion-extension stress to mouse LF led to macrophage infiltration in the progression of LF hypertrophy. Although both in vivo and in vitro studies have demonstrated that mechanical stress can be a potential stimulus factor in LF hypertrophy, the mechanism of hypertrophic LF progression still remains unknown [8].

P16 is a negative regulator of the cell cycle progression. Liu [9] studied the expression and interaction of cyclin D1 and p16 in fibroblasts of pathologic scars and the result showed that p16 could suppress the excessive cell proliferation. Zainuddin A1 [10] found γ-Tocotrienol could prevents cell cycle arrest in aged human fibroblast cells through inhibiting p16 (INK4a) pathway. These findings suggest that P16 plays an important role in decelerating fibroblast cell cycle progression, cell proliferation and apoptosis, and may be associated with degenerative hypertrophy of the LF.

The S100 protein is a calcium-binding protein that functions in cell growth, differentiation, cell cycle regulation, apoptosis, and cell surface receptor activity [11]. S100 plays an important role in the development of inflammatory skin diseases, autoimmune diseases and cardiovascular diseases [12]. S100 is expressed specifically in a small group of cells including macrophages, microvascular endothelial cells, fibroblasts and keratinocytes during inflammation and oxidative stress [13, 14]. Zhong suggested that S100A8 and S100A9 can promote fibroblast activation and fibrosis in the dermis [15]. Since the progression of LF hypertrophy is always accompanied by inflammation and tissue fibrosis, we thereby speculate that S100 may also play a key role in the LF hypertrophy.

This study plans to study three groups of LF including simple lumbar disc herniation (LDH), single-segment LSS (SLSS) and double-segment LSS (DLSS). The purpose of the study is to explore: (1) the histological difference of LF hypertrophy in three groups; (2) Correlation between the degree of LF hypertrophy assess through MRI and hematoxylin-eosin (HE) and Verhoff’s Van Gieson’s (VVG) staining; (3) whether P16 and S100 are associated with LF hypertrophy; (4) Whether SLSS and DLSS differ in P16 and S100 expression.

## Methods

### Specimens collection

The research program was approved by the Institutional Review Committee of Tianjin Union Medical Center, and all procedures were performed according to the Declaration of Helsinki. All patients received written informed consent before operation. The LF was collected during surgery after obtaining the informed consent from patients. This protocol was approved by the institutional ethic boards of Tianjin Union Medical Center. Thirty LF specimens were obtained from 30 patients who had undergone decompressive laminectomy for neurogenic claudication to SLSS and DLSS. Thirty LF specimens were obtained from 30 patients in LDH patients during surgery. The baseline data between each group has no significant difference (*p* > 0.05, Table 1). All the patients had been unresponsive to conservative measures for at least 3 months. None of these patients receive selective nerve-root blocks. Patients who had isthmic and degenerative spondylolisthesis, scoliosis, or fractures were excluded from this study. We collected the entire layer of the LF and removed all the epidural fat from the LF specimens. Each specimen was fixed in 4% neutral formalin, decalcified with 20% ethylenediaminetetraacetic acid (EDTA) for 4-6 weeks and then embedded in paraffin for histologic and immunohistochemical analysis.

**Table 1:** Baseline Data

Group	Cases number	gender		Age (years, mean ± SD)	Disease course (d,mean ± SD)
		male	female		
LDH	30	16	14	42.9 ± 12.6	92 ± 16.3
SLSS	30	15	15	43.5 ± 10.6	88 ± 12.9
DLSS	30	14	16	45.2 ± 11.8	95 ± 10.6
Stastic value		*χ*2 = 0.926		*F* = 0.286	*F* = 0.758
*p* Value		> 0.05		> 0.05	> 0.05

### MRI measurement

MRI examination was performed before operation. MRI T1 phase cross-section measurement was performed by hospital PACS system in Tianjin Union Medical Center. The thickness of the LF was compared in each group as proposed by Fukuyama [16] .The thickness of the LF was measured from the mid-point of the LF to the ventral side of the inner rim. The lumbar spinal canal oblique diameter is measured from the midpoint of the dorsal side of the ligamentum flavum to the midpoint of the posterior margin of the vertebral body. The relative thickness (RT) (%) of LF is calculated as the percentage of LF thickness compared to lumbar spinal canal oblique diameter. Three independent measurements from 3 surgeons were averaged to determine the RT of an individual sample.

### Histologic analysis for elastin degradation and fibrosis of the LF

Two consecutive sections (4 μm thickness) were obtained and stained with HE and VVG stain, respectively. HE stain was used to characterize the LF collagen deposition and VVG stain was used to characterize the elastic fiber. Histologic analysis was independently performed by 3 pathologists on 10 randomly selected, high power fields (× 400) images of each sample.

The HE stained slides were independently evaluated and graded according to LF elastin degradation. Grade 0 indicates normal tissue which shows no elastin degradation region. Grade 1 indicates that elastin degradation is < 25% of the entire area. Grade 2 indicates between 25 and 50% elastin degradation. Grade 3 indicates a 50 to 75% elastin degradation and grade 4 indicates > 75% elastin degradation.

Scores were assigned to each VVG stained slide based on the presence and morphology of the LF elastic tissue. The following grading criteria were used: 0, normal; 1, short fragmented elastic fibers; 2, intermediate between 1 and 3; 3, fibrillar elastic fibers; 4, intermediate between 3 and 5; and 5, absent or nearly absent.

All HE and VVG stained slides were viewed using an Olympus BX50 light microscope (Olympus Corp), and digital images were taken at × 200 magnification with an Olympus DP20 microscope camera. The image files were saved as high-resolution tag image file format files. Images were captured from dural and dorsal aspects regions of the LF.

### Immunohistochemical analysis for the localization of P16 and S100

The LF specimens were fixed in 10% neutral formalin and embedded in paraffin. 5 μm thick sections were collected, dewaxed in xylene, and rehydrated in graded ethanol solutions. Sections were then incubated with purified mouse monoclonal antibody specific to P16 (Maixin Biotechnology, Fuzhou, China, 1:100) or purified mouse monoclonal antibody specific to S100 (Maixin Biotechnology, Fuzhou, China, 1:100). The positive controls were also performed according to the manufacturer’s recommendation. A routine immunoperoxidase staining technique using 3,3-diaminobenzidine tetrahydrochloride was performed.

### Statistical analysis

The results of the absolute and relative thickness, and the histological ratings of HE and VVG stained slides of the three groups were compared using one-way ANOVA. We determined the relationships between the thickness and the histological ratings of HE- and VVG-stained slides using Pearson’s correlation coefficient test. Data are shown as mean ± SD, and a *p* value less than 0.05 was used to determine statistical significance. The IBM SPSS Statistics version 21.0 software (IBM, New York, NY, US) was used for all analysis.

## Results

### MRI measurement

In total, 90 LF measurements was taken and analyzed. Their absolute and relative thickness values are presented in Table 2. Absolute and relative LF thicknesses were greater in the DLSS group compared with the SLSS and LDH groups (*p* <  0.05). The mean thickness in the DLSS group was 5.658 mm (RT = 43.107), compared with the 4.924 mm (RT = 36.520) and 2.886 mm (RT = 21.330) in the SLSS and LDH groups.

**Table 2:** Absolute and relative thickness of LF in the LDH, SLSS and DLSS groups

		LDH	SLSS	DLSS	*F*	*p*
LF thickness	Absolute (mm) ± SD	2.886 ± 0.592	4.924 ± 0.843	5.658 ± 0.793	73.270	< 0.01
	Relative (%) ± SD	21.330 ± 5.532	36.520 ± 8.686	43.107 ± 7.996	44.024	< 0.01

*LF* ligamentum flavum, *LDH* lumbar disc herniation, *DLSS* double-segment lumbar spinal stenosis, *SLSS* single-segment lumbar spinal stenosis

Relative thickness formula: (LF thickness/lumbar spinal canal oblique diameter) × 100; Means in percents ± standard deviations

### Histologic Stud*y*

**Fig. 1:**
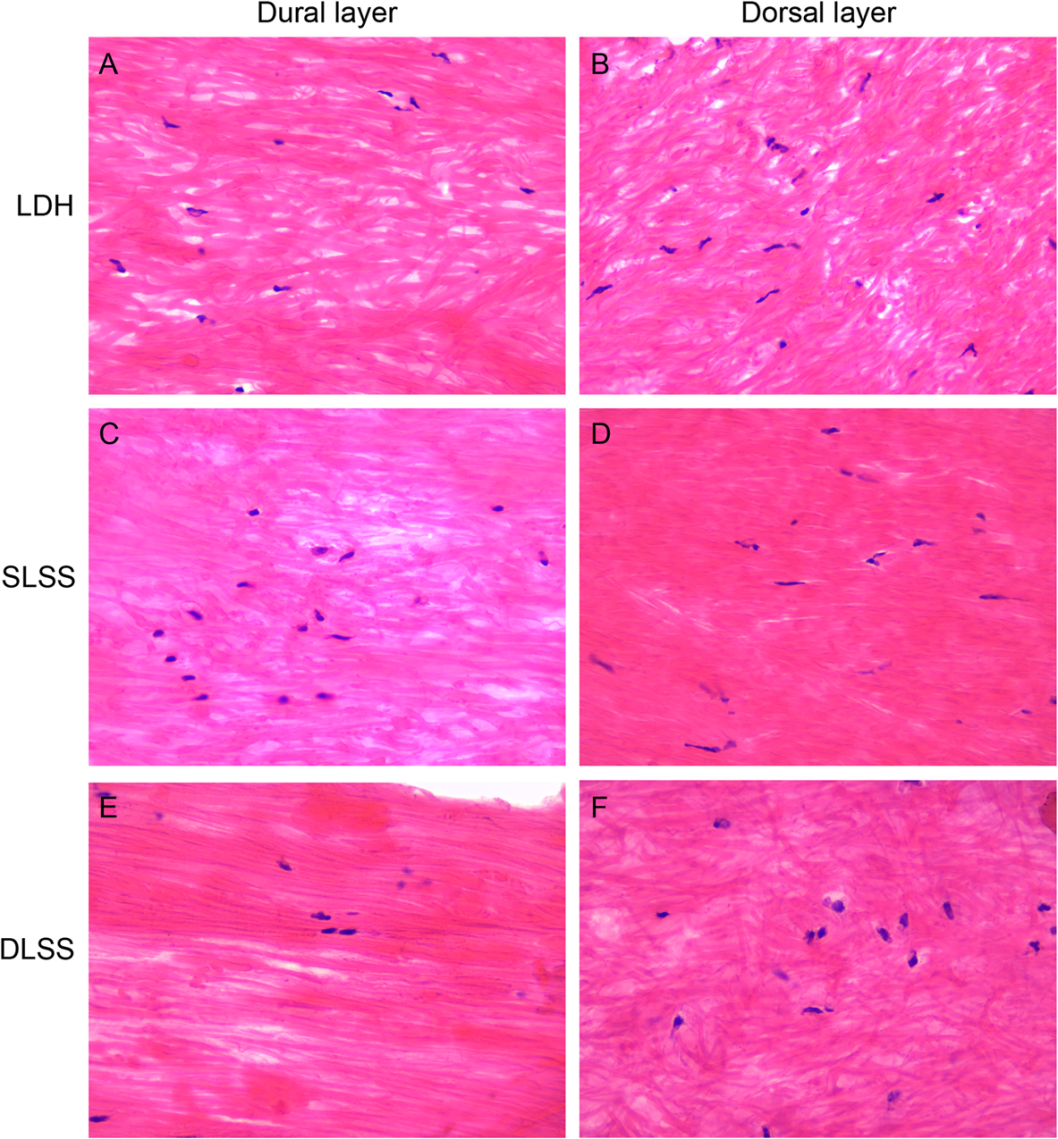
There was no significant difference between the dorsal aspect and the dural aspect in the LDH group for HE stained (**a** and **b**). There is a significant increase in positive HE stained elastic tissue from dorsal aspect to the dural aspect in SLSS (**c** and **d**) and DLSS groups (**e** and **f**)

**Fig. 2:**
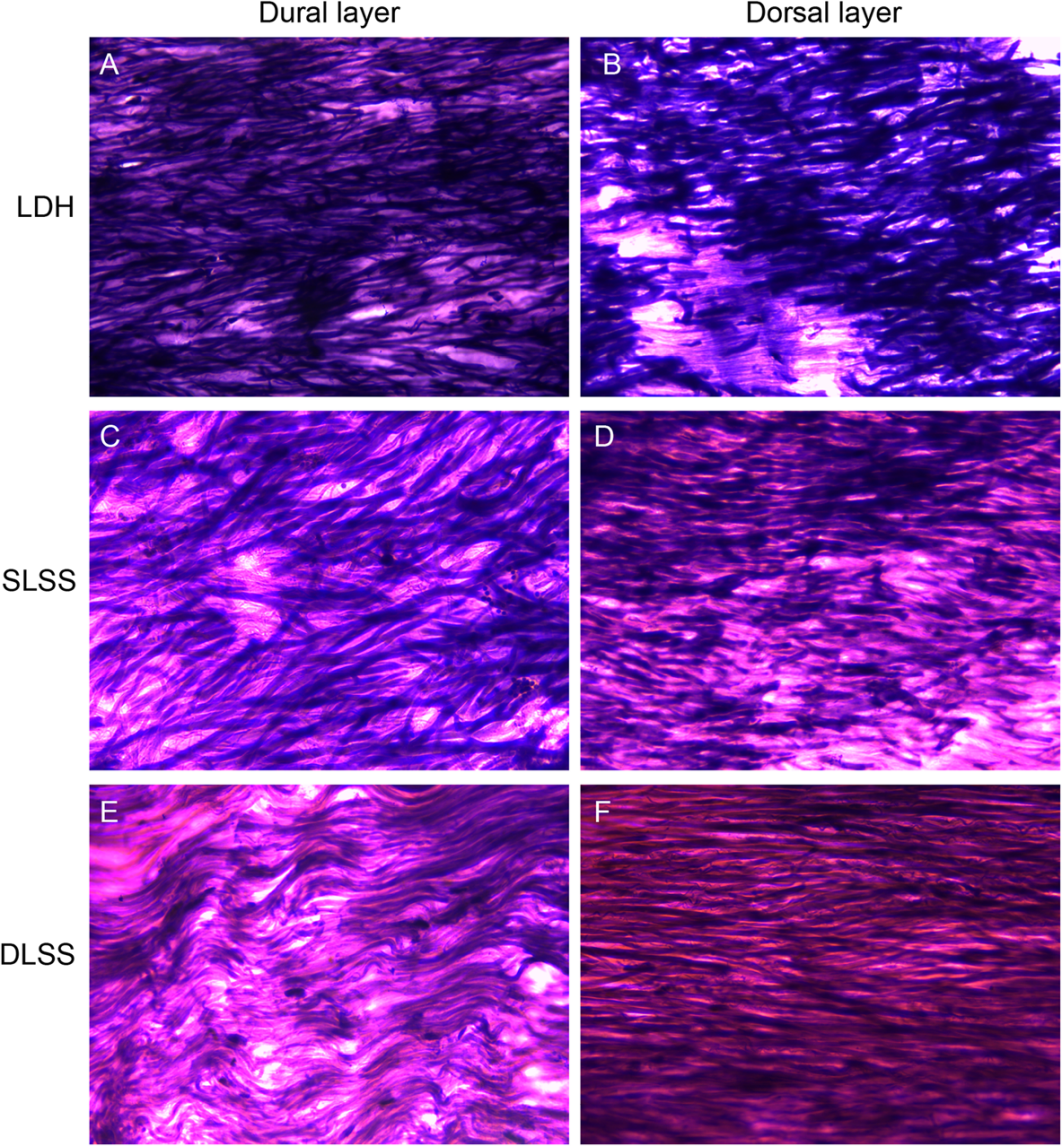
There was no significant difference between the dorsal aspect and the dural aspect in the LDH group for VVG stained (**a** and **b**). A significant decrease in positive VVG stained elastic tissue was seen in the dorsal aspect compared with the dural aspect in the SLSS (**c** and **d**) and DLSS groups (**e** and **f**)

HE staining showed a significant increase of elastic tissue from the dorsal aspect to the dural aspect in SLSS and DLSS groups (Fig. 1). VVG staining showed significantly less elastic tissue in the dorsal aspect compared with the dural aspect in the SLSS and DLSS groups (Fig. 2). There was no significant difference between the dorsal aspect and the dural aspect in the LDH group in both HE and VVG staining.

The HE and VVG stained slides were histologically rated. The result is shown in Table 3. DLSS group was rated higher compared with the SLSS and LDH groups (*p* <  0.05) in the HE assay. The mean rate for the DLSS group was 2.850, while the mean rate for the SLSS and LDH groups was 1.950 and 0.600. In the VVG staining assay, a greater rate was seen in the DLSS group compared with the SLSS and LDH groups (*p* <  0.05).

**Table 3:** Histological ratings of H&E- and VVG-stained slides in the LDH, SLSS and DLSS groups

		LDH	SLSS	DLSS	*F*	*p*
Histological rating	H&E stain, Mean ± SD	0.600 ± 0.598	1.950 ± 0.826	2.850 ± 0.875	42.625	< 0.01
	VVG stain, Mean ± SD	1.050 ± 0.759	2.250 ± 0.851	3.550 ± 1.050	39.041	< 0.01

*LDH* lumbar disc herniation, *DLSS* double-segment lumbar spinal stenosis, *SLSS* single-segment lumbar spinal stenosis

There was a significant correlation between HE mean rating and relative LF thickness in the DLSS group (*r* = 0.562; *p* = 0.010; Table 4). No significant correlation was seen in other groups.

**Table 4:** Correlation between H&E rating, VVG rating and absolute LF thickness, relative LF thickness in the LDH, SLSS and DLSS groups

		H&E rating		VVG rating	
		*r*	*p*	*r*	*p*
LDH	Absolute LF thickness	0.004	0.986	0.050	0.835
	Relative LF thickness	−0.233	0.323	0.086	0.718
SLSS	Absolute LF thickness	−0.205	0.387	0.159	0.502
	Relative LF thickness	−0.090	0.706	−0.128	0.592
DLSS	Absolute LF thickness	0.375	0.103	−0.163	0.492
	Relative LF thickness	0.562	0.010	0.182	0.442

*LF* ligamentum flavum, *LDH* lumbar disc herniation, *DLSS* double-segment lumbar spinal stenosis, *SLSS* single-segment lumbar spinal stenosis

### Immunohistological study

**Fig. 3:**
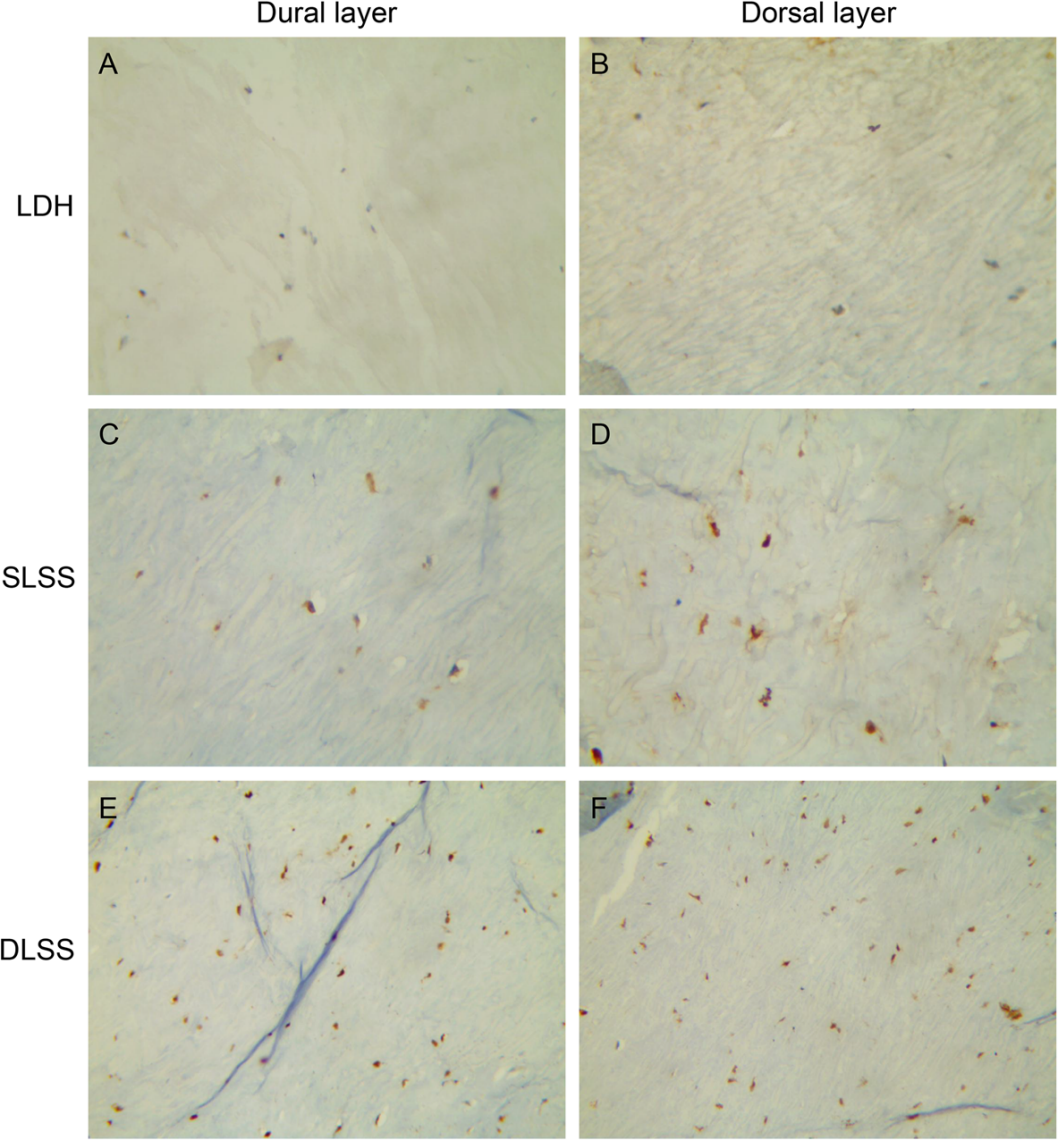
The LDH group showed negative staining of P16 in both the dural and dorsal layers (**a** and **b**). Partially positive staining of P16 were showed in the SLSS group (**c** and **d**). The specimens in the DLSS group showed positive staining of P16, especially in the dorsal layer(**e** and **f**)

**Fig. 4:**
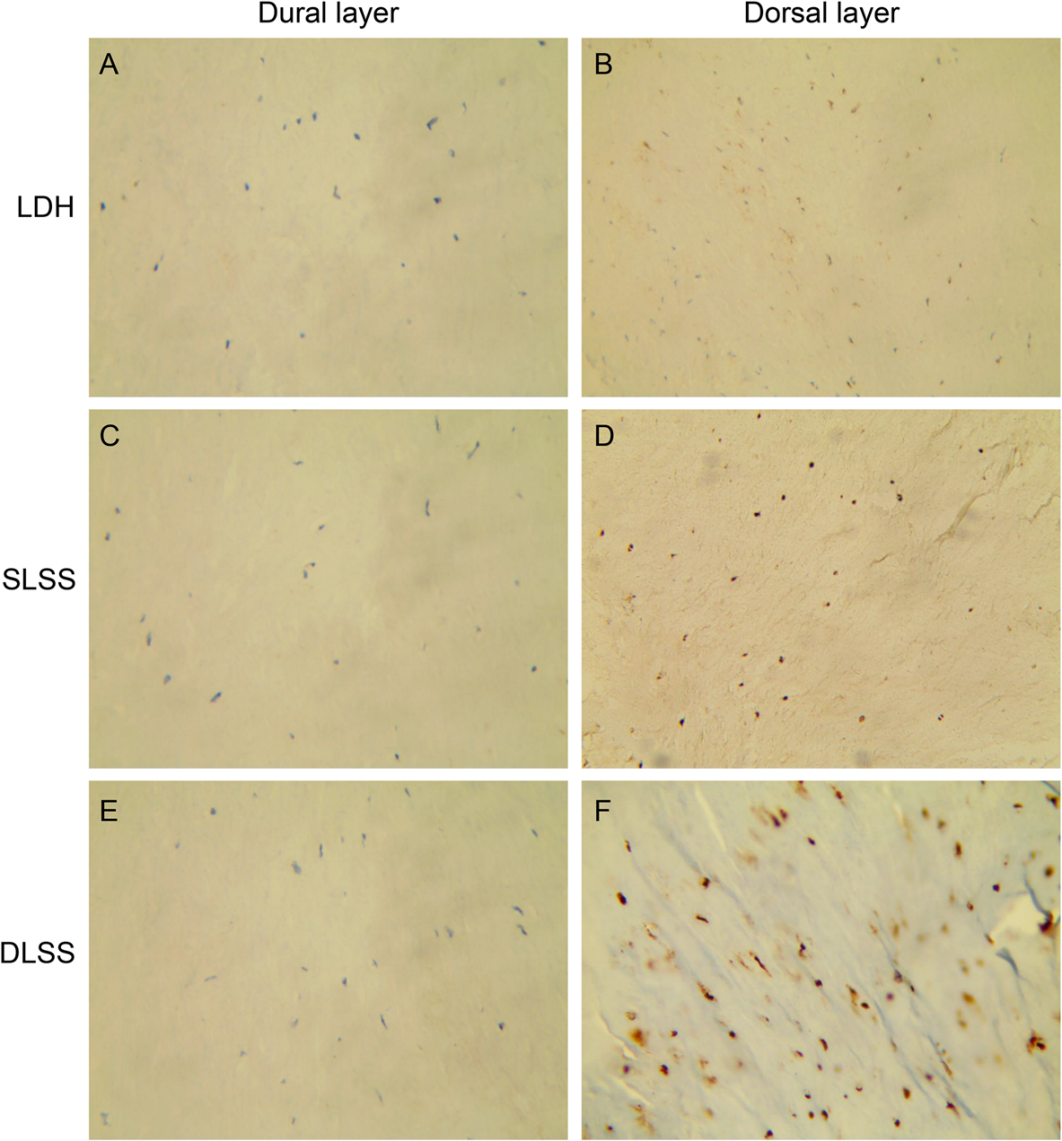
For the LF specimens in the three groups, S100 staining was absent in the dural layer (**a**, **c** and **e**) and were stained in the dorsal layer (**b**, **d** and **f**)

DLSS group were positively stained for P16, especially in the dorsal layer. Almost all samples in the SLSS group were partially positive for P16. The LDH group were P16 negative in both the dural and dorsal layers (Fig. 2). All three groups were S100 positive in the dorsal layer of the LF. However, S100 staining was absent from the dural layer of the LF in the three groups (Figs. 3 and 4).

## Discussion

MRI plays an important role in the diagnosis and treatment of lumbar spinal stenosis. Sakamaki [17] measured the thickness of LF at lumbar spine using MRI. No correlation between the thickness of LF and the decrease of the disc height in elderly patients was suggested in his study. LF thickness increased with age especially at L4–5 and L3–4 and this increase maybe started between age 30–39 at L4–5. Therefore, in this study, we use L4–5 as the measured segment. Kim [3] designed the morphological parameter as the measurement of LF by MRI which may be closely related to the symptom of LSS. The results indicate that the LF area and the thickness of the LF are equally important for the clinical treatment of LSS. We determined the resection area of the LF based on the MRI examination before surgery, and the removed LF was divided into the dural and dorsal aspect. The MRI measurement showed that the absolute and relative LF thicknesses were greater in the DLSS group compared with the SLSS and LDH groups. The reason may be that LF thickness is related to the lumbar stress and DLSS patients can potentially stand more stress than the patients in SLSS and Simple LDH groups [16]. With the development lumbar spine degradation, the stress of the L4/5 segmental LF is greater and the LF hypertrophy maybe aggravated.

The LF is an important connecting structure at the back of the spine. It is mainly composed of elastic fibers, collagen fibers, reticular fibers and matrix. Yabe demonstrated through Elastic-Mason staining and alcian blue staining that degenerative LF possess reduced elastic fibers and elevated proteoglycans levels compared to normal LF [5, 18]. Our study found a significant increase in positive HE and VVG stained elastic tissue from the dorsal aspect to the dural aspect in LSS group. During LSS develop process, LF hypertrophy progressed from the dural aspect to the dorsal aspect. Our result suggests that this dural to dorsal progression may be related to the greater stress in the dorsal aspect of LF. Therefore, preventing the deterioration of elastic fibers in LF and controlling the number of fibroblasts may be the key to prevent or reverse the hypertrophy of LF.

Kosaka [19] discovered the pathomechanism of LF hypertrophy in the aged population. His study indicated that elastic fibers decreased and collagenous fibers were mainly accumulated in the dorsal aspect during LF hypertrophy. Sairyo [20] found that the dorsal aspect of the LF was always subjected to hypertrophy than the dural aspect due to the accumulation of mechanical stress with lumbar motion development or aging. In our study, the dorsal layer showed the most severe fibrotic changes in the VVG staining. The dorsal aspect is subjected to greater tensile force than the dural aspect in flexion due to its attachment to the lamina. The histologic results showed that fibrosis appeared in all LSS hypertrophic LF. The elastic fibers became thin and fragmented while collagen grew into diffused and massive especially.

Usually, the pathological process of LF hypertrophy involves mechanical stress, inflammation, angiogenesis and fibrosis occurs sequentially [21].The mechanical compression may lead to changes of inflammatory cytohines, nitric oxide and cyclooxygenase [22].Usually the longer the duration of these symptoms are, the more likely thickened LF occurrs during this process [23].In our histologic study, a higher HE rate was detected in the DLSS group compared with the SLSS and LDH groups (*p* <  0.05). A greater rate was also seen in the DLSS group in the VVG staining compared with the SLSS and LDH groups. A significant correlation between HE mean rating and relative LF thickness in the DLSS group was also established in this study. (*r* = 0.562; *p* = 0.010). Given pathological process mentioned above, we hypothesize that SLSS and DLSS may have different pathological mechanisms for the development of LF hypertrophy. Our finding also suggests that LF may respond to LSS and LDH differently and show different pathologic properties in the LSS and LDH.

Sairyo [24] showed that LF hypertrophy is due to the accumulation of inflammation-related scar tissure. Various growth factors or cytokines play important roles in the pathological process. However, the mechanism is still unclear. Pittozzi [25] evaluated the effect of resveratrol on the senescence-associated secretory phenotype (SASP) and on adhesion-related processes in cultured human MRC5 fibroblast. He found that p16(INK4a) protein production was changed when inflammation occurred. Honma [26] investigated the inflammation-related metabolic abnormalities in fasting rat, S100 was increased transcriptionally in peripheral leukocytes, Previous study showed that P16 is a negative regulator of cell cycle and plays an important role in the development of pathological scars [9]. Scar formation and LF hypertrophy both involved the proliferation of fibroblasts. S100 protein level has been shown to be elevated in hypertrophic skin fibroblast during scar formation, thus we suspect that P16 and S100 may be involved in the process of LF hypertrophy.

In the immunohistochemical analysis, the LDH group was P16 negative. Partially positive staining of P16 were observed in the SLSS group. The specimens from the DLSS group showed positive staining of P16, especially in the dorsal layer. This result indicated that the fibrogenic process might occur mainly on the dorsal side of the LF and P16^+^ fibroblasts is accumulated in the dorsal layer during inflammation [27], We need to investigation by means of western blot and real-time PCR in the biologic study and culture fibroblasts in vitro to confirm this hypothesis. S100 staining was absent from the dural layer and were observed in the dorsal layer in all groups. This result suggested that dorsal aspect may experience more mechanical stress than the dural aspect. However, although the LF hypertrophic process and scar formation process both involve fibrosis, the progression of LF hypertrophy can also largely influenced by the mechanical stress. These differences can potentially explain why we did not see significant changes in the S100 level LF hypertrophy.

One of the limitations of this study is that increased expression of P16 and S100 was observed in LF Immunohistochemical analysis. We should carry out biologic study, including Western Blot analysis and Real-Time Polymerase Chain Reaction to further clarify the mechanism of LF hypertrophy according to S100 and P16. In addition, for the sake of LF hypertrophy prevention and treatment strategy, inhibition studies on S100 and P16 can be performed. Further investigation on how S100 and P16 regulate fibroblasts proliferation might also provide important clues for the future treatment of LF hypertrophy.

## Conclusions

We found there was more severe elastin degradation and fibrosis of the LF in the DLSS patients compared with the SLSS and LDH patients. Increased expression of P16 and S100 was associated with LF fibrosis and thickening, suggesting that the expression of P16 may related to the LF hypertrophy in the patients who suffer from LSS. LF hypertrophy process may not be associated with high expression of S100.

## Data Availability

The datasets used and analyzed during the current study are available from the corresponding author on reasonable request.

## Abbreviations

DLSS: Double-segment LSS
EDTA: Ethylenediaminetetraacetic acid
HE: Hematoxylin-eosin
LF: Ligamentum flavum
LSS: Lumbar spinal stenosis
RT: Relative thickness
SLSS: Single-segment LSS
VVG: Verhoff’s Van Gieson’s
